# Gut–Brain Inflammation and Disrupted Homeostasis Due to Activation of Mast Cells and Microglia

**DOI:** 10.3390/ijms27041768

**Published:** 2026-02-12

**Authors:** Pejman Katiraei, Richard E. Frye, Theoharis C. Theoharides

**Affiliations:** 1 Wholistic Kids and Families, Santa Monica, CA 90403, USA; pkatiraei@wholisticminds.com; 2 Autism Discovery and Treatment Foundation, Phoenix, AZ 85050, USA; drfryemdphd@gmail.com; 3 Institute for Neuro-Immune Medicine, Dr. Kiran C. Patel College of Osteopathic Medicine, Nova Southeastern University, Ft. Lauderdale, FL 33328, USA; 4 Laboratory of Molecular Immunopharmacology and Drug Discovery, Department of Immunology, Tufts University School of Medicine, Boston, MA 02111, USA

**Keywords:** brain, flavonoids, folinic acid, gut, inflammation, luteolin, mast cells, microbiome, microglia, toxins

## Abstract

Recent data from the Centers for Disease Control (CDC) indicate that the incidence of Autism Spectrum Disorder (ASD), a neurodevelopmental disorder characterized by deficits in social communication and the presence of restricted interests and repetitive behaviors, has increased to 1 in 31 children. Individuals with ASD have a constellation of neurological, behavioral, sensory, feeding, gastrointestinal, and immunological issues. Even though there is some genetic component to the pathogenesis of ASD, accumulation of environmental and pathogenic toxins could contribute to disruption of the gut–blood-barrier (GBB) and blood–brain barrier (BBB) via activation of mast cells (MCs) and microglia, resulting in a chronic cycle of gut–brain inflammation. Here we discuss how various environmental, pathogenic, and stress factors can disrupt gut–brain homeostasis to create susceptibility and epigenetic effects that contribute to the development of ASD. We also suggest simple ways to address some of the key pathogenetic processes involved in ASD.

## 1. Introduction

Autism Spectrum Disorder (ASD) is a complex neurodevelopmental disorder characterized by deficits in social communication and the presence of restricted interests and repetitive behaviors [1,2,3,4,5]. Individuals with ASD have a constellation of neurological, behavioral, sensory, feeding, gastrointestinal, and immunological issues. ASD is increasing at an alarming rate and now impacts 1 in 31 children in the United States [6], with a projected total cost of $461 billion by 2025 [7].

No single evidence-based pathway can explain all the different types of ASD [2,8]. Genetic vulnerabilities are known to be a significant contributing factor to ASD [3]. In particular, both susceptibility genes and epigenetic processes have been considered [9,10,11,12]. ASD is likely due to genetic vulnerabilities activated by environmental toxins and stressors [13,14]. Environmental factors, particularly environmental toxicants, may also significantly contribute to the increased prevalence of ASD [15,16]. Some of these vulnerabilities occur at the gut level [17], including how host genetics interact with the gut microbes to shape the immune and metabolic state of ASD [18].

The microbiome can influence immune homeostasis [19] and the gut–brain axis, thus contributing to neuroinflammation in degenerative diseases [20] and ASD [21]. Environmental toxins, including herbicides such as glyphosate, heavy metals, synthetic compounds and plastics, mold toxins, air pollution, viruses such as SARS-CoV-2, and excessive use of antibiotics, are just some examples of factors that can compromise gut permeability, induce gut-mediated inflammation, and disrupt the microbiome [7,22,23,24,25,26,27,28]. Maternal autoimmune conditions and infections are also known to disrupt the microbiome [29] and may contribute to an increased risk of ASD [30,31,32].

This paper discusses how such exposures disrupt the gut–brain axis, leading to a chronic state of neuroinflammation primarily via activation of mast cells (MCs) and microglia that can explain the physical, cognitive, psychological, and social findings in at least a subset of individuals with ASD. This review further stresses the importance of neuro-immune interactions in inflammatory responses [33] and allergic diseases [34,35], especially in immune-mediated gastrointestinal (GI) disorders [36] and neurodegenerative diseases [20,37] such as ASD.

## 2. Gut Microbiota, Gastrointestinal Issues, and Neurologic Health

Increasing evidence supports a link between microbiota disequilibrium and the gut–brain axis [38,39] in a number of neuropsychiatric and neurodegenerative disorders [20,39,40,41,42,43,44,45,46,47], including ASD [48], possibly via induction of neuroinflammation [49]. However, significant gaps exist as to how gut–brain dysfunction contributes to the pathogenesis of ASD, even though neuroimmune interactions have been considered [50,51,52] and the gut microbiome may induce epigenetic alterations [53].

Children with ASD are at a 500% higher risk of developing feeding problems [54], such as food selectivity, food refusal, and poor oral intake, as compared to neurodevelopmentally normal children [55,56]. Children with ASD have texture aversion and strong preferences for foods like carbohydrates and processed foods [57,58] and a higher risk of healthy food avoidance: vegetables (56% refusal), eggs (43%), fruits (42%), chicken (35%), and meat (24%) [56]. These dietary preferences can exacerbate any microbiome disruption [59] and increase the abundance of *candida* [60]. The high amounts of carbohydrates and fats, and few dietary fibers, can also dramatically enhance the absorption of bacterial lipopolysaccharide (LPS) and induce further inflammatory responses [61].

Individuals with ASD are four times more likely to have GI symptoms compared with healthy controls [22,57], including constipation, diarrhea, and abdominal pain [62]. These symptoms can begin as early as 6–18 months of age [63]. Also, individuals with ASD have a significantly higher prevalence of intestinal inflammation and inflammatory bowel disease [64], abnormal intestinal permeability [65], abnormal microbiome makeup [66,67] and possibly a higher prevalence of *candida* versus controls [68].

Gut microbiota are implicated in autoimmune [69] and inflammatory conditions [69]. Imbalances in the microbiome and GI tract can then lead to a disrupted gut–blood-barrier (GBB) (80). With respect to subjects with ASD, there are significant alterations in gut microbiota [70] with a shift towards depletion of beneficial species and an increase in pro-inflammatory species [71,72,73,74,75]. These changes are characterized by elevations of some microbial-derived metabolites [76], especially short-chain fatty acids (SCFAs), with reduced levels of butyrate [77,78] and increased levels of propionic acid [78,79]. Propionate has been associated with the development of ASD-like behavior in animal models [80,81,82,83,84,85,86], possibly by inducing neuroinflammation [87,88] and disrupting synaptic communications [89,90]. Instead, butyrate has protective effects [91,92,93,94,95]. Unfortunately, propionate is a food preservative [96,97,98] that can exogenously increase gut levels of propionate. It was also reported that valeric acid, another gut-derived SCFAs, acts as a selective inhibitor of class I histone deacetylase 3 (HDAC3), contributing to epigenetic effects [99].

There can also be elevations in the production, toxicity, and systemic absorption of other toxins and metabolites. For example, 3-(3-hydroxyphenyl)-3-hydroxypropionic acid (HPHPA) is a gut microbial metabolite that is also a neurotoxin. The p-cresol microbiome produced a metabolite that influences noradrenaline production and dopamine metabolism. Indole and 3-methylindole are other gut microbiome-produced metabolites that can interfere with serotonin metabolism [100].

ASD individuals also have complex patterns of systemic immune dysregulation [101,102], with brain inflammation involving microglia activation [103] and elevations of various cytokines in the cerebrospinal fluid (CSF) [102,104], including the neuropilin disruptor matrix metalloproteinase-9 (MMP-9) [105]. The immune dysregulation often presents as higher rates of food allergies [106], as well as atopic diseases including asthma, allergies, and eczema, which are also strongly correlated with a higher risk of ASD [106,107]. It is interesting that there is now evidence of a skin-gut axis involving microbiota in the development of allergies [35,52,108,109] (see Section 4 later).

Individuals with ASD have abnormal neuronal apoptosis [110], myelination [111], and neuroplasticity [112]. They also have dysfunction in multiple areas of cognition, including: attention, executive functioning, working memory, praxis, and motor planning [113]. Individuals with ASD experience significantly higher rates of anxiety [114,115], obsessive–compulsive disorder (OCD) [116], abnormal perception of fear [117], anger, and aggression [118]. This chronic state of psychological stress can trigger inflammation [119] within the GI tract via the release of corticotropin-releasing hormone (CRH), leading to disruption of both the gut–blood-barrier (GBB) [120,121,122,123,124,125,126] and the blood–brain-barrier (BBB) [127,128,129] through activation of MCs, which can also release CRH on their own [130].

As many as 80–95% of subjects with ASD also struggle with difficulties in processing complex sensory information [131]. The severity of these sensory processing disorders is a significant predictor of ASD severity and everyday functioning [132,133,134]. This complex constellation of findings that are associated with ASD may be due to disruptions within the GI tract and microbiome. Increasing evidence indicates that sensory neuroimmune interactions affect the integrity of barriers [135].

## 3. Gut Dysbiosis Contributes to Chronic Gastrointestinal Inflammation and Dysfunction

The GI tract harbors trillions of bacteria, fungi (the mycobiome), viruses, LPS, and dietary antigens. Such triggers (Table 1) could lead to disruption of the GBB and BBB, leading to chronic neuroinflammation. Separating this complex and potentially deadly “internal” gut content from our circulation and other organ systems, including the brain, is a tightly regulated interface consisting of a mucus layer, a single epithelial layer held together by tight junction proteins (e.g., zonulin and occludin), immunoglobulin A, defensins, and a carefully orchestrated immune surveillance in the lamina propria of the gut [136]. As long as this GBB is intact and the gut bacterial and fungal communities are healthy, the likelihood of systemic and chronic inflammation is low; otherwise, the likelihood of chronic systemic and nervous system inflammatory responses rises dramatically [137,138,139,140].

Disruption of the GBB allows pro-inflammatory and neurotoxic compounds to circulate systemically and reach the brain via a disrupted BBB. These processes result in neuroinflammation, which affects neurotransmitter balance and neural network function, ultimately contributing to behavioral and cognitive symptoms characteristic of ASD.

Abnormalities in T-cell activity directly impact microbial diversity [141], leading to a deviation of the commensal population from a healthy, diverse symbiotic profile to microbial communities with reduced complexity and over-representation of particular taxa of microbes [136,142].

The innate immune system within the gut has pattern recognition receptors (PRRs) whose primary purpose is to detect pathogens, including the commensal bacteria, by recognizing pathogen-associated molecular patterns (PAMPs) [143]. LPS is among the most potent pro-inflammatory neurotoxins [144,145]. The toxicity of the LPS increases with the degree of dysbiosis [145]. Gut dysbiosis and a disrupted gut barrier allow for the translocation of LPS through the gut lining into the systemic circulation, allowing the development of low-grade, chronic, generalized toxin-associated effects [146], as is found in individuals with severe ASD [147]. Numerous animal models suggest a strong link between generalized toxin effects and the features of ASD [148,149,150,151]. One study in rats reported ASD features after a single prenatal exposure to LPS [152].

Once absorbed, LPS can trigger systemic inflammation, with a reduction in T regulatory (Tregs) lymphocytes and an increase in Th17 and Th1 lymphocytes, along with increased TNF-α, NFκB, IL-6, IL-8, IL-10, and IL-12 [145,153]. Children with ASD also have activated inflammasome complexes, including the NLRP3 inflammasome [154], which are complex systems that play a critical role in the regulation of the body’s inflammatory response. LPS can rapidly prime and activate this inflammasome [155], which could be further primed by candidalysin [155,156]. Furthermore, within the intestinal lining, LPS binds to toll-like receptor4 (TLR4) on intestinal cells [146,157,158], thus triggering additional inflammation and further disrupting the GBB. Commensal bacteria can promote the migration of MCs in the intestine [159]. MCs could have a protective role against enterobacteria via TLR-4 [160]. Viruses have also been associated with ASD, and they can also stimulate MCs via TLR-9 [161].

Peripheral gut inflammation and damage can activate the microglia [162,163]. In healthy adult volunteers, 1 ng/kg of LPS can trigger robust microglial activation in most areas of the brain as measured by positron emission tomography (PET) scans within three hours of injection [153]. In rodents, a single intraperitoneal injection of 5 mg LPS/kg causes microglial activation that persists for at least 12 months [153]. Several other animal studies have also shown that systemic LPS can activate the microglia [164,165,166,167]. If animals are given multiple doses of 1 mg LPS/kg (over several days), a model for chronic generalized toxin effects, they experience neuroinflammation, BBB permeability, and rapid neurodegeneration [153], findings that are common in individuals with ASD [110,168].

Even low levels of LPS can induce sickness behavior through the elevation of inflammatory cytokines [153,169,170,171]. Sickness behavior, an adaptive change in behavior as a result of inflammation, has been described in individuals with ASD and can present as anxiety, appetite loss, depression, headache, impaired alertness and focus, lethargy, muscle pain, and social withdrawal [153,172].

The impact of LPS on the CNS is likely through multiple mechanisms, including the vagus nerve [163]. LPS cannot pass through or directly disrupt the BBB^115^. On the other hand, histamine, various toxins, cytokines, a high-fat diet, as well as a high-sugar diet are just a few of the factors that can compromise the BBB [173,174,175,176,177], thus allowing LPS to directly enter the CNS. Stress via the release of CRH can also disrupt the BBB through activation of MCs [127,128,129].

**Figure 1:**
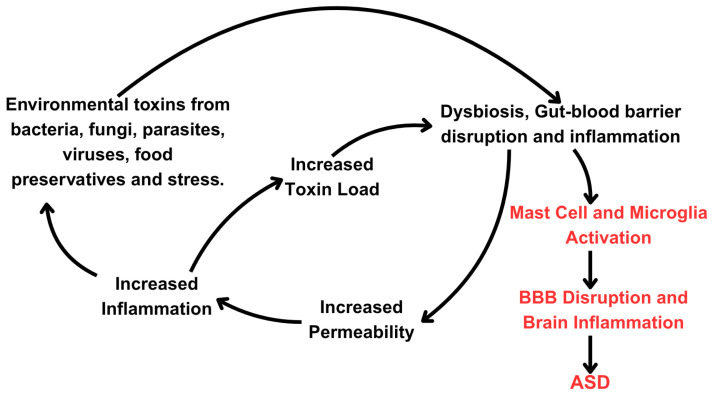
Diagrammatic representation of the proposed events involving toxin-activated mast cells leading to gut–brain chronic neuroinflammation, microglia activation, and disruption of neuronal connectivity and homeostasis. The compounded effects of various environmental toxins and physiological stressors induce intestinal permeability and inflammation, which may allow the involvement of secondary inflammatory triggers, including various microbial compounds, to induce a secondary gut and brain inflammatory response in the presentation of ASD.

Gut inflammation can be linked to ASD symptoms in several ways. Changes in the balance of bacteria within the microbiome lead to the overactivation of the immune system, resulting in the release of inflammatory cytokines (such as IL-6, IL-8, and TNF-α). This chronic inflammation can compromise the GBB, leading to increased intestinal permeability, often referred to as “leaky gut” (Figure 1). Furthermore, certain toxins, like mycotoxins, can alter the production of intestinal-specific immunoglobulins [178], which then significantly influence the composition and behavior of the bacterial and fungal makeup of the microbiome [141,179,180,181,182]. Gut microbiome disruptions can also induce *candida* species to switch from a harmless commensal to a virulent pathobiont, which is then able to invade tissues and disseminate in the body [183,184,185]. Further complicating this picture, *candida* can alter the makeup of the microbiome through multiple mechanisms [186] and prevent the regrowth of *lactobacilli* after antibiotic treatment while promoting the colonization of *enterococcus* [184]. The invasive form of *candida* and the toxin its hyphae form secretes, candidalysin [187], can stimulate significant MC activation [188,189], thus creating a vicious cycle of chronic gut and immune dysfunction. These mycobiome abnormalities can influence the maturation and priming of the immune system [190] and exacerbate the allergic state [191] that, as noted, is more prevalent in ASD [192]. A recent paper reported that certain uremic toxic signatures, termed “metabolic index of gut dysfunction (MIGD),” could help stratify phenotypes of subjects with ASD and different stool patterns [193].

These findings may explain microbiome and mycobiome abnormalities found in individuals with ASD [21,67] with complex patterns of dysbiosis that are still being studied, including abnormalities in *clostridium* species [70].

Microglia shape neuronal plasticity and connectivity and synaptic function by maintaining proper wiring through neuronal pruning [194,195,196]. Microglia regulate myelin growth and integrity [197] and, when activated, may cause severe demyelination [198]. They are also involved in abnormal neuronal apoptosis [199]. Moreover, by disrupting neuronal circuitry, microglial activation impairs the processing and integration of various sensory and emotional responses as part of the presentation of ASD [102,200,201]. Microglial activation [202] has been documented in ASD [203], confirmed through postmortem findings [204] and on functional positron emission tomography [205].

Microglial activation alters the neuronal pathways of the amygdala, which has been shown to disrupt the fear threshold and may present as ASD [206]. Disruptions in the amygdala can also play a significant role in the pathogenesis of aggressive behavior [207], addictive behaviors [208,209], anxiety disorders [210], impulse control disorder [211], attention deficit disorder (ADD) [212], depression, and a host of other neuropsychiatric findings [213], all of which are all noted in individuals with ASD [114,115,117,118,214]. Activated microglia may induce post-synaptic calcium elevation, causing increased neuronal reactivity and disrupting glutamate signaling [196]. Microglia can also alter the levels of quinolinic acid [215], a potent neurotoxin implicated in ASD [216]. Microglial activation can also disrupt the behavior of the astrocytes [217], which in turn can further disrupt glutamate homeostasis, upset GABA regulation, and neuronal pruning [196].

Beyond the effects of the microglia, human and animal studies have shown that endotoxin-induced inflammation can also increase the neural responses in the anterior cingulate cortex and prefrontal regions, further impacting the processing of social and emotional information [218].

As such, each individual has a genetically determined threshold of gut resilience and tolerance to various stressors or toxins. Thus, if an environmental factor or combination of factors supersedes this threshold, the GI tract and microbiome can become compromised by setting off a complex cascade of gut-immune-brain dysregulation. At the center of this dysregulated axis are the GI MCs (Figure 1).

Beyond these microbial metabolites, a host of other triggers can also enter the brain, induce neuroinflammation, and disrupt neuronal connectivity (Table 1). In particular, a number of environmental risk factors have been associated with ASD [219]. These include endocrine disruptors [220,221], sodium sulfide [222], the most common herbicide glyphosate [223,224,225], heavy metals [226], and food pollutants [227,228].

## 4. Molecular Aspects of Mast Cell Activation and Gut Integrity

The human leukocyte antigen (HLA) haplotypes can influence the sensitivity to environmental triggers such as mold (Table 2), determine the specificity of T lymphocyte and natural killer (NK) cell responses, as well as influence the makeup of the commensal bacteria [229]. The HLA haplotypes A2, DR4, and DR11 have been found to create major susceptibility for ASD [101,230,231]. The presence of these haplotypes may alter monocyte populations in children with ASD and GI symptoms [232].

There is a large population of MCs in the GI tract [233], where they play a fundamental role in maintaining the intestinal barrier by regulating epithelial function and integrity, managing the defensive and immunoregulatory function, modulating both innate and adaptive mucosal immunity, and maintaining neuro-immune interactions [233]. These functions all play a fundamental role in the health or disease of the gut [102,234]. Many of the almost 300 MC-derived mediators (Table 3) can have profound effects on the integrity of the GBB and BBB, permitting the entry of toxic molecules into the blood and then into the brain, thus leading to neuroinflammation. Activation of GI MCs was shown to increase GBB permeability in mice [235]. In addition, skin MCs can also influence gut microbiota [236,237,238]. It is not surprising that mast cell activation syndrome (MCAS) is associated with gut dysfunction [239].

Mast cells are typically distinguished based on their secretory granule content of proteolytic enzymes [240] as “mucosal” MCs (MMCs) that contain only tryptase and “connective tissue” MCs (CTMCs) that contain both tryptase and chymase [241,242,243,244,245,246,247]. Additional evidence indicated that there may be more than one form of chymase [248]. Ms can also be distinguished based on the granule content of biogenic amines [249]. However, MCs can assume different phenotypes [250]. MCs can also be further distinguished based on their expression of the low-affinity Mas-Related G-Protein Coupled Receptor Member X2 (MRGPRX2). Apparently, skin and synovial MCs, but not lung and heart MCs, expressed this receptor [251]. Similarly, MCs in human nasal polyps also did not express MRGPRX2 [252]. Furthermore, seven MC subsets were identified based on unique transcriptome signatures, of which MC1+ were found in the bladder, MC2+ in the lungs, and MC4-MC7+ in the skin [253]. Intestinal MCs were recently better characterized using single-cell transcriptomics and showed that different MC subpopulations express different genes [254,255]. In humans, MMCs (present in lamina propria) contain only tryptase, while CTMCs (in the submucosa) contain tryptase, chymase, carboxypeptidase 3 (Cpa3), and cathepsin. MMCs originate from fetal hematopoietic stem cells, while CTMCs apparently originate from the yolk sac and can maintain themselves independently. Moreover, a transcriptomic profile obtained by single-cell RNAseq analysis revealed that MCs express specific gene products, most prominently vascular endothelial growth factor (VEGF. In particular, MMCs were characterized by high expression of genes encoding for *Mcpt1* and *Mcpt2* proteases, for adhesion molecules (*Itgae*, *Itga2a*, *Ly6e*), and for the chemokine receptor Cxcr1, whereas CTMCs express genes for *Cma1*, *Mcpt4*, *Tpsb2*, and *Cpa3* proteases, for the chemokine *Ccl2*, lipid metabolism genes (*Apoe*), and especially *Mgbrb2* genes [254].

Mast cell phenotyping may be further modified depending on the expression of other mediators [256] and surface receptors [257], as well as the presence of cytokines in the microenvironment [258]. The specific function or reactivity of the different MC subgroups is not well understood. For instance, MCs purified from normal foreskin varied greatly in their expression of FcεRI and in their release of histamine in response to an allergic trigger [250,259].

MCs are typically activated by allergens crosslinking specific immunoglobulin E (IgE) bound to its high-affinity receptor, FcεRI [260,261]. The results are rapid exocytosis (degranulation) of several pre-stored mediators [262,263]. In addition to FceRI, MCs express many more surface receptors activated by different stimuli [264,265], including non-allergenic triggers [266,267,268,269,270,271] such as complement fragments [272,273], eosinophilic cationic protein, and platelet-activating factor (PAF), which is both released from and stimulates MCs [274,275] and eosinophils [276,277,278] and thus contributes to allergies [279]. PAF also induces IL-6 production [280,281,282], which in turn stimulates the production of PAF [283,284]. MCs also respond to many neuropeptides [264], such as CRH [263], nerve growth factor (NGF) [285], neurotensin (NT) [286], substance P (SP) [287,288], and the SP-related hemokinin-1 (HK-1) [289], which have pro-inflammatory properties [290,291,292,293,294] via activation of high-affinity receptors or the low-affinity receptor MRGPRX2 [295,296,297]. Activation of MCs by cationic peptides and drugs via MRGPRX2 [298,299] could lead to an effect additive to that of FceRI [300].

Mast cells can also synthesize CRH [130], HK-1 [289], NGF [49], NT [301], SP [302], and other neurotrophins [303]. Moreover, SP could induce the ST2 receptor for the alarmin IL-33 [304], further exacerbating MC release of pro-inflammatory molecules, especially when primed by IL-33 [270,305]. Moreover, there appears to be some form of interaction between MRGPRX2 and FcεRI, leading to amplification of MC activation [306].

Mast cells are the sentinels of our immune system [307] and can be activated by many of the same triggers that disrupt the GBB [233,308,309,310], including heavy metals, herbicides (e.g., glyphosate), polychlorinated biphenyl (PCB), LPS, mycotoxins, as well as pathogens [308,311,312], including *Helicobacter* pylori [313], via activation of toll-like receptors (TLRs) [160,314,315,316,317]. Once activated, MCs orchestrate complex arrays of immune activation leading to allergic inflammation, findings common in individuals with ASD [102,318].

Mast cells can also be stimulated via activation of TLRs [314,315,317,319,320,321] by toxins [322] and pathogens [323,324,325], such as viruses [308,326], including severe acute respiratory syndrome coronavirus 2 (SARS-CoV-2) [327,328,329,330,331,332,333]. In fact, TLRs have been implicated in neurodegenerative diseases [334,335,336,337,338].

Upon stimulation, MCs store, synthesize, and secrete as many as 350 mediators [339], while MCs can also take up, store, and release another 50 or so [340,341] affecting multiple organs (Table 3). Some of the best-known preformed mediators include chymase, heparin, histamine, tryptase, and tumor necrosis factor (TNF) [342]. Histamine has been the main mediator associated with MCs [343], but it is also released from basophils [344]. Tryptase is almost exclusively secreted from MCs, especially during anaphylaxis and systemic mastocytosis [345,346], as well as in many cases of mast cell activation syndrome (MCAS) [347]. Tryptase activates the pro-inflammatory protease-activated receptors (PARs) and generates anaphylatoxins (C3a, C5a) [348]. MC granules can also store tissue remodeling enzymes, such as carboxypeptidase A3 (CPA3) and matrix metalloproteinases (MMPs) [241,349,350,351].

Mast cells also secrete newly synthesized mediators 6–24 h after stimulation (late-phase reaction) [331]; these include leukotrienes, prostaglandin D_2_ (PGD_2_) [352], cytokines (IL-5, IL-6, IL-31, IL-33, and TNF-α) and chemokines (CCL2, CCL5, and CXCL8) [353,354,355], and VEGF, which is released without tryptase [356] and was shown to be elevated in the serum of patients with mastocytosis [357,358].

It is of interest that the alarmin IL-33 [270,305,359] stimulates MCs, significantly increasing the ability of SP to stimulate the release of VEGF [287,360], IL-31 [361], TNF-α, and IL-1β [304], as well as CCL2 and CCXL8 [362] and other newly synthesized mediators from human MCs [270]. Murine MCs secrete and respond to IL-33 [363], but IL-33 makes MCs unresponsive to bacterial cell wall products [364]. Mast cell-derived IL-1β can then stimulate MCs to release IL-6 selectively without degranulation [365,366]. IL-6 is elevated in patients with mastocytosis [367,368,369] and with COVID-19 [370,371]. In fact, IL-6 promotes MC proliferation [372] and is constitutively released in the presence of the D816V-KIT mutation [373].

Mast cells also communicate with T-cells during the immune response [374,375]. In fact, MC can function as antigen-presenting cells [376] and induce the maturation of dendritic cells [377]. Mastcells can also act as antigen-presenting cells to activate T cells [102,378,379,380]. Mast cells can be induced to express HLA-DR, especially when stimulated by interferon γ (ING-γ) [381] secreted during viral [308,312] and bacterial or fungal [382,383] infections.

Aberrant MC activation can cause profound disruption to the gut through damage to these tight junction proteins via mediators such as histamine, MMP-9, TNF-α, and tryptase (Table 3), resulting in increased intestinal permeability and inflammation, a critical step that allows the translocation of the commensal bacteria [136,183,318]. Through the release of numerous mediators (Table 3), Mast cells can further diminish the integrity of the GBB, sensitize dendritic cells to microbial signals, including LPS, and influence the behavior of the innate and adaptive immune response [384,385,386].

Changes to the GBB and microbiome are sufficient for the pathogenesis of food allergies [387]. Especially in young children, even minor changes to the barrier function early in life can lead to exposure to luminal antigens, which can result in allergies in later stages of life [388]. These gut abnormalities can also explain the GI symptoms such as abdominal pain, constipation, and diarrhea found in individuals with ASD [389,390,391]. Small intestinal mucosal damage may also decrease the activity of diamine oxidase (DAO) [392], a key enzyme that degrades histamine, and thus exacerbate the detrimental effects of histamine that is released by activated MCs. In fact, it was definitively shown that food allergies release histamine that sensitizes GI sensory nerve endings, resulting in the sensation of abdominal pain [391,393].

Investigation of MC numbers or evidence of degranulation may be missing the point, as MCs can release specific mediators via differential release mechanisms [394] such as serotonin [365,366,395] or IL-6 [365,366] or VEGF [356] without degranulation, but rather via intragranular changes [396]. MCs can also release the content of individual granules [397] or via “piece-meal degranulation” [398], granule-associated vesicle transport [399], or the release of extracellular vesicles [400,401,402,403,404,405,406].

## 5. Mast Cells and ASD

Mast cells serve as an “immune gate to the brain” [129] and activate microglia in a two-way communication, leading to neuroinflammation [407,408,409,410]. Increasing evidence now implicates neuroinflammation in ASD [411]. Gut inflammation activates MCs within the nervous system [412]. This is further impacted by psychological stress [206,413]. Stress during gestation has been associated with an increased risk of developing eczema [414,415] and ASD [206,416,417,418,419,420,421,422]. Family history of ASD was strongly associated with the severity of ASD in the offspring [416]. In fact, prenatal stress was reported to “rewire” the gut–brain axis via long-term effects on microbiota, the intestinal barrier, and hippocampal inflammation [423]. A recent paper reported that eczema at birth originates from dysregulated MCs in utero [424].

Immune dysregulation, especially during gestation, has repeatedly been implicated in ASD [101]. Numerous studies have shown that there is a strong statistical association between maternal atopic conditions, such as asthma and eczema, and ASD [415,425,426,427,428], as well as between atopic conditions in the offspring, especially eczema, and ASD [102,107,429,430,431,432]. Moreover, there was an association between early-life gut microbiome, lifestyle factors, and the development of eczema [433]. A recent paper reported that maternal stress could trigger early-life eczema via fetal MC programming [424]. A genome-wide pleiotropic study showed a strong relationship between eczema and neuropsychiatric disorders [434]. Moreover, prenatal allergic inflammation in rats altered communication in brain regions important for cognitive and social behavior [435] and also “rewired” the gut–brain axis [423].

**Figure 2:**
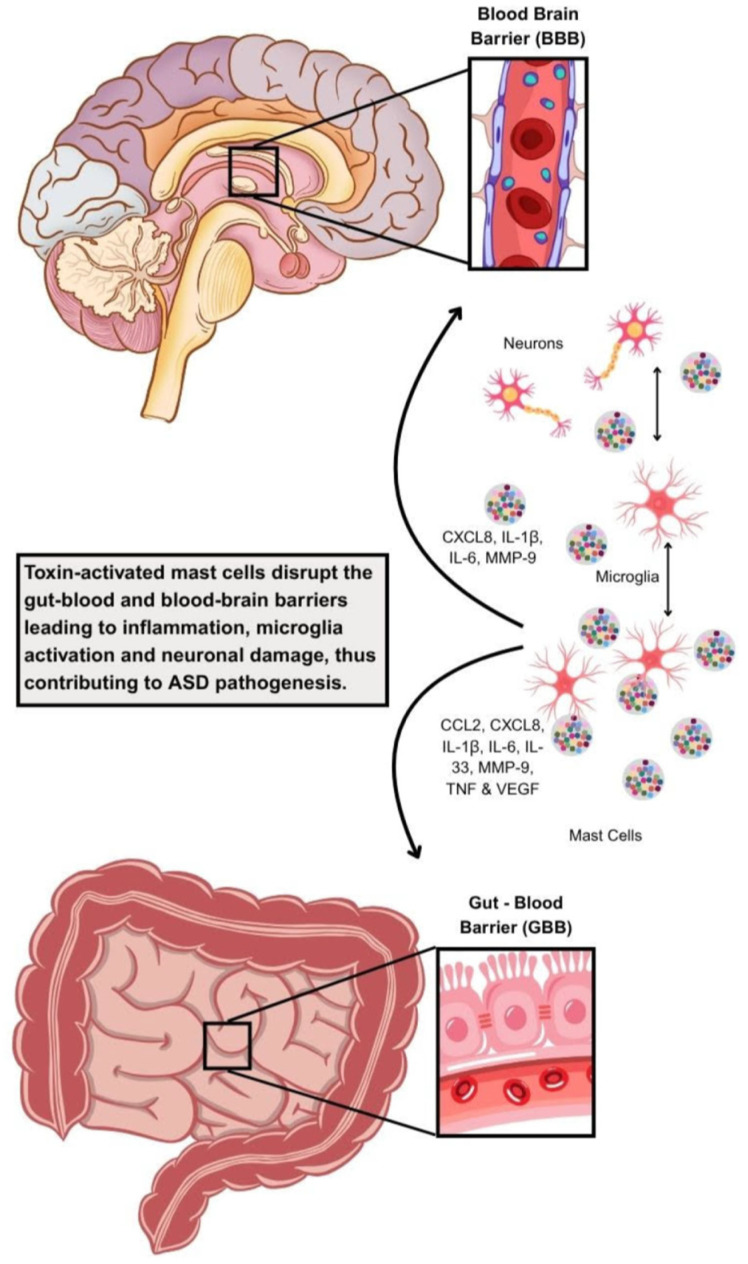
Diagrammatic representation of how toxin-activated mast cells disrupt the gut–blood and blood–brain barriers, as well as activate microglia and disrupt neuronal connectivity, leading to neuroinflammation and ASD.

Mast cell activity is intimately tied to microglial activity, and the activation of one cell line can lead to the activation of other immune cells through multiple pathways (Figure 2) [217].

The role of MCs and microglia in individuals with ASD has been well documented [102,436,437,438]. Persistent or aberrant activation of MCs may disrupt the BBB via release of multiple chemokines (e.g., CCL2, CXCL8), cytokines (e.g., IL-1β, IL-6, IL-17, IL-33, TNF-α), tissue disruptors (e.g., chymase, MMP-9, tryptase), and neurotoxic (e.g., CRH, histamine, osteopontin, PGD_2_, TNF−α) molecules (Table 3), and may create localized inflammation in the area of the basal ganglia that disrupts neuronal connectivity and contributes to ASD-related behaviors [439]. Mast cell-induced neuroinflammatory response can also utilize additional mechanisms [102,103,436]. Mast cells can also undergo mitochondrial translocation to the cell surface with the extracellular secretion of mitochondrial nucleic acids that are then detected by the immune system as ‘innate pathogens’, triggering a significant inflammatory response, potentially contributing to ASD [440]. In fact, mitochondrial DNA was identified in exosomes derived from ASP patients and could stimulate cultured human microglia [441]. Mitochondrial DNA may also induce a neuro-inflammatory response, which has been found to alter behavior in mouse models [442]. These different neuro-inflammatory responses could significantly contribute to ASD in some individuals [439].

Gut-mediated MC activation can increase histamine levels within the CNS [443]. Mast cells within the brain produce over 50% of all brain histamine [444]. In animal models, intraperitoneal LPS injection can activate brain mast cells and cause a rapid elevation of central histamine [445]. Histamine plays a critical role in modulating the nervous system [446]. It regulates alertness and is also a key wake-promoting neurotransmitter that influences the circadian rhythm and sleep–wake behavior [444]. Sleep disruption is a common feature of ASD [447]. Elevated levels of central histamine can also disrupt the vestibular system, which is critical for balance, motor planning, and sensory perception [446]. Histamine has also been shown in animal models to directly or indirectly influence various sensory pathways: sound processing [448], tactile sensation [449], and olfactory perception [446,450]. The disruption of these sensory pathways may explain the host of sensory findings found in individuals with ASD, including eating problems and food texture avoidance [451]. The intensity of sensory issues has been associated with more significant social difficulties, lower adaptive functioning, and lower or divergent visual exploration of social environments in children with ASD [452].

## 6. Suggestions on How to Diagnose and Address Gut–Brain Inflammation

Unfortunately, there are profound limitations in our current diagnostic tools to detect the critical pathophysiological processes discussed above in individuals with ASD.

Nevertheless, there are a number of useful biomarkers (Table 4) that reflect chronic inflammation and gut–brain axis dysfunction. For instance, levels of serum IgG4, which are associated with food intolerance, increased in ASD [453,454]. Elevated stool histamine, eosinophilic cationic protein, and calprotectin are reasonable indicators of gut allergic and inflammatory processes. Urinary N-methylhistamine, leukotriene E_4_, and prostaglandin F_2a_ (must be collected cold in 24 h urine) reflect activation of GI MCs.

While it is beyond the scope of this paper, a number of options are available to address parts of this proposed cycle of gut dysfunction and endogenous toxicity (Table 5). Modulating the gut microbiota is a reasonable approach [455]. Nutraceuticals are increasingly used in neuropsychiatric disorders [456].

Probiotics have been proposed for neurologic disorders [457] and ASD [458]. In particular, Bifidobacteria, especially adolescents/brevis/infants/longum [91,459,460], are preferred because they degrade histamine, along with butyrate [91,94], berberine, and lactoferrin that could provide additional benefits and may reduce the risk of ASD [461], as they all have both antibacterial and anti-inflammatory properties [462,463,464,465,466,467]. Many studies have also shown that vitamin D3 deficiency is an independent risk factor for ASD [468,469,470,471]. Supplementation with vitamin D3 in pregnant mice reduced expression of IL-6 and IL-17a in the fetal brain and ileum of mice [472] and contributed to gut health [473].

Certain fruits and vegetables that contain histamine (e.g., avocado, cheese, pineapple, sardines, sauerkraut, spinach, and tomatoes) should be best avoided, especially in those with polymorphisms in the histamine-degrading enzymes diamine oxidase (DAO) and histamine N-methyl transferase (HNMT) [474]. DAO is a naturally occurring enzyme within the gastrointestinal tract and is responsible for the degradation of histamine within the gut. As many as 40% of individuals have DAO polymorphisms, reducing their ability to degrade gut histamine. Moreover, intestinal mucosal damage may decrease the DAO activity [392]. Histamine intolerance has also been associated with anxiety disorders [475]. Exogenous DAO supplementation can significantly reduce histamine levels within the gastrointestinal tract and minimize the signs and symptoms of histamine intolerance [476], including extra-intestinal symptoms such as headaches [476]. To our knowledge, DAO enzymes are safe and well-tolerated. However, DAO preparations vary considerably in their stated activity, and most of the enzyme will be degraded by the acidity of the stomach unless they are in acid-resistant formulations.

Polyphenolic compounds have been shown to accumulate in the gut and render beneficial actions [477]. Such compounds have been reported to target the TLR pathway [336,478]. A number of recent reviews have stressed the potential importance of flavonoids in ASD [479,480] by inhibiting inflammation [481,482] or inhibiting gut microbiota [483]; however, it should be kept in mind that many individuals with ASD have phenol intolerance, and gene analysis should be conducted for the enzymes catecholamine-ortho-methyl transferase (COMT), monoamine oxidase (MAO), and phenol sulfur transferase (PST) that catabolize phenolic compounds.

**Figure 3:**
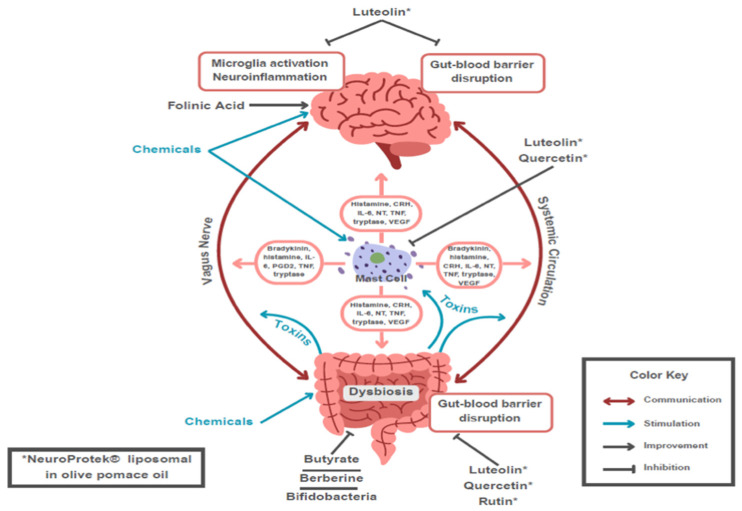
Diagrammatic representation of how mast cell-derived mediators contribute to gut–brain inflammation via disruption of the gut–blood and blood–brain barrier, and showing the key targets of intervention by selecting natural molecules.

**Figure 4:**
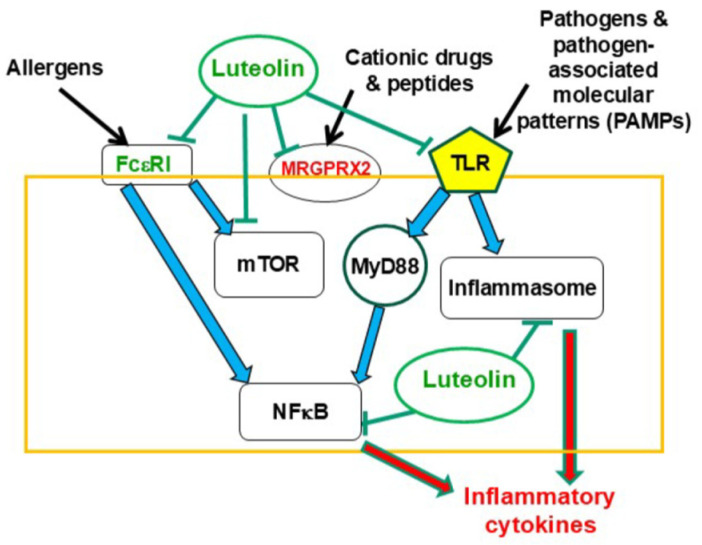
Diagrammatic representation of points of action of luteolin and other flavonoids. Black arrows indicate stimulation; blue arrows indicate activation of downstream events; red arrows indicate secretion of mediators; green T lines indicate inhibition.

The flavonoid luteolin (tetrahydroxyflavone) is a well-studied bioflavonoid with a host of anti-inflammatory properties [484,485,486]. Luteolin has been found to inhibit mast cell and T cell activation [487] and decrease levels of histamine and TNF-α [488,489]. In fact, luteolin was recently shown to be a more potent inhibitor than the “mast cell stabilizer” drug cromolyn [490]. Luteolin also has protective effects against activation of the nuclear factor kappa-light-chain-enhancer of activated B cells (NFκB) in intestinal macrophages [491] (Figure 3). Luteolin can also enter the brain and reduce microglial activation [492], particularly as a result of LPS [493], as well as have antibacterial properties [494]. Luteolin further protected against propionate-induced organ damage and ASD-like behavior in animal models [495,496]. Figure 4 is a schematic representation of the points of action of luteolin and other flavonoids.

The structurally related quercetin (pentaxydroxyflavonol) can also inhibit MCs and is more effective than cromolyn at blocking mast cell cytokine release [497]. Quercetin can also balance the Th1/Th2 immune response [498], reduce gut permeability while improving microbial diversity [499], mitigate propionate-induced behavior in a rat model of ASD [500], and protect against LPS-induced gut damage through multiple mechanisms [501]. Rutin is the quercetin glycoside, and it is important because of its ability to liberate quercetin in the gut. Recent publications reported that luteolin protects against neuronal injury [502] and experimental colitis in mice [503] by inhibiting activation of the NLRP3 inflammasome.

Significant clinical improvements of symptoms in children with ASD were demonstrated from the use of a dietary supplement (NeuroProtek^®)^, which contains (in softgel capsules, >95% purity) liposomal (formulated in olive pomace oil) luteolin (100 mg/softgel), quercetin (70 mg/softgel), and the quercetin glycoside rutin (30 mg/softgel) [489,504,505]. Due to the fact that about 20% of children have phenol intolerance (increased irritability when eating chocolate, berries, or strawberries), NeuroProtek Low Phenol^®^) was developed to contain reduced amounts of quercetin (40 mg/softgel) and rutin (1 mg/softgel) and is now available in liquid form (with and without natural lemon flavor), allowing the dropper to deliver it under the tongue for better absorption. The noted studies all suggest that these supplements are safe and well-tolerated.

The delivery form of the flavonoids is important because in powder form they are absorbed less than 10% from the gut; instead, olive pomace oil used in NeuroProtek^®^) not only increases absorption from the gut via the creation of liposomes that contain the flavonoids, but it also offers the well-known cytoprotective properties of olive oil [506]. Unfortunately, many cheaper preparations of luteolin and quercetin in powder form do not disclose the source, are of low purity, and/or the daily dose requires multiple capsules or tablets [507]. The common notion that if you take higher amounts of luteolin or quercetin in powder form, it will eventually allow some of the flavonoids to be absorbed is not only wrong but dangerous, as the unabsorbed flavonoids accumulate in the gut and disrupt the microflora [508].

The structural luteolin analog, tetramethoxyflavone (methoxyluteolin), has no phenol groups and is even more potent than luteolin in inhibiting both mast cells and microglia [288,362,509,510,511]. Methoxyluteolin has been incorporated in the novel anti-allergic skin lotion (GentleDerm^®^) [512], which is particularly useful in those individuals with both eczema and ASD (Table 5).

One additional compound that may be helpful in ASD is palmitoyl ethanolamide (PEA), a naturally occurring fatty acid amine found in soybean lecithin, egg yolk, and peanut meal. PEA has noticeable anti-inflammatory properties and can regulate MC activation [513,514,515] by reducing the release of TNF-a and histamine [516]. Furthermore, PEA displays neuroprotective properties and can inhibit microglial activation [517], particularly in response to LPS exposure [518]. In two case reports, PEA was found to be beneficial in ASD [518] (Table 5).

Addition of folinic acid (calcium folinate, Leucovorin) has been shown to significantly improve brain health, cognition, and language by bypassing surface folate receptors and the enzyme methylenetetrahydrofolate reductase (MTHFR), especially in those with anti-folate receptor antibodies and MTHFR polymorphisms (Table 5) [519].

## 7. Limitations

The present manuscript is a narrative review. There is no singular trigger, event, or genetic or physiological process that is solely responsible for the onset of ASD in the majority of cases [520].

The effects of gut–brain inflammation are not necessarily limited to the pathogenesis of ASD but could be implicated in other neuropsychiatric disorders. However, unique combinations of triggers appear to be more relevant to ASD and should be addressed. This review excludes the metabolic, neurochemical, and other pathophysiological findings that are associated with ASD. Nevertheless, the role of mast cell and microglial activation and their triggers within the gut compounds in ASD are important areas for further research. Moreover, the interventions presented are only suggestive and based mostly on the collective empirical experience of the authors. The clinical efficacy of the compounds discussed may be limited in the face of significant gastrointestinal disease or serious environmental exposures, such as living in a home with severe mold contamination.

## 8. Future Directions

Efforts should be made to develop noninvasive ways to assess:

- Endotoxemia—There is no commercially available diagnostic tool available to directly assess endotoxemia.
- Total toxin load—Currently, only specialty tests are available to assess select categories of toxins, and their results are at times called into question.
- Mycobiome—There are many commercially available stool kits to assess the bacterial component of the microbiome, but they lack the sensitivity to accurately detect disturbances of candida or other fungal components.
- Mast cell activation. Serum histamine has a half-life of less than two minutes and thus cannot accurately detect histamine imbalances. Serum tryptase can be used to assess significant MC burden (e.g., systemic mastocytosis), but its ability to detect MC activation within the gut or brain.
- Microglia activation—There is no commercially available diagnostic tool available to assess microglia activation.

There should also be research to study key microbiota-MC-microglia interactions by developing a human organoid [521] gut–brain-on-a-chip model [522,523,524]. Our efforts would be to develop a human organoid gut–brain-on-a-chip model containing gut epithelial cells, endothelial cells, MCs, and microglia derived from individuals with ASD and normotypic controls.

It is the total load of pathogenic and environmental toxins, which varies from individual to individual, compounded by other infectious [4] physiological and psychological stressors that may be responsible for the onset of ASD in some individuals.

We believe that in these individuals, there is a moment during gestation/delivery, in infancy, or in early childhood where the gut barriers, microbiome, and gut-mediated immune responses surpass a threshold of homeostasis [15] and enter into a perpetual cycle of neuroinflammation, ultimately involving activation of mast cells [525] and microglia [526,527]. The natural interventions suggested could inhibit some of the pathogenetic pathways and allow the gut–brain axis to recover [496,528].

The molecular events involved in the gut–brain axis disruption should be a major focus of research, but unfortunately, there are no reliable functional models for ASD [529]. An exciting possibility would be to generate human-induced pluripotent stem cell (hiPSC)-derived organoids [530,531] in a gut–brain axis chip [523,524] to study the proposed interactions. Such organoids containing microglia were recently used to study early-life immune challenges [532] and showed a reduced number of GABAminergic neurons [533,534].

## 9. Conclusions

While there is sufficient data to identify some of the factors contributing to ASD risk, additional research is needed to bring this vast array of findings into one cohesive model that has the power to assess the unique exposome for each individual and appreciate the total physiological impact it can have on ASD. Urgent research is needed to assess the role environmental and pathogenic toxins may play in the development of ASD and their epigenetic effects. Development of effective inhibitors of MC activation would be useful, especially in those children with ASD and MC-related comorbidities.

## Figures and Tables

**Table 1:** Key environmental, pathogenic, and pro-inflammatory triggers.

Affecting the Gut–Brain Axis
Bacterial endotoxins (e.g., lipopolysaccharide = LPS from *E. coli*); Bacterial neurotoxins (e.g., *Clostridium botulinum*); Bacterial exotoxins (e.g., *Staphylococcus aureus*); Bacterial byproducts (e.g., propionic acid from the *Bacteroidetes phylum*); Candidalysin; Cytokines (e.g., IL-1β, IL-6, IL-8, TNFα); Food preservatives (e.g., parabens, propionic acid, sodium nitrite, sulfites); Heavy metals (e.g., aluminum, mercury); Herbicides (e.g., atrazine, glyphosate); Microplastics (e.g., in beer, seafood); Mold (mycotoxins, especially Ochratoxin A = OTA); Parasites (e.g., Giardia lamblia cysteine proteases that damage gut mucosa); Propionic acid; Quinolinic acid; Neuroendocrine disruptors (e.g., Bisphenol A = BPA); Valeric acid; Viruses (e.g., rotavirus nonstructural protein 4 = NSP4).

**Table 2:** HLA-DR/DQ haplotypes.

Haplotype	Genes Involved	Associated Health Risks
7-3-53	HLA-DRB107, DQB103, DRB401 *	Increased susceptibility to mold biotoxins.
17-2-52A	HLA-DRB117, DQB102, DRB301 *	Impaired toxin clearance leading to chronic inflammation and CIRS.
18-4-52	HLA-DRB118, DQB104, DRB301 *	Linked to heightened sensitivity to environmental triggers such as mold.
HLA-DRB1 * 13	HLA-DRB113 *, DQ6, DRB3	Increased frequency in mold-sensitive individuals with asthma.
HLA-DRB1 * 03	HLA-DRB103 *	Increased frequency in individuals with mold hypersensitivity.
HLA-DQB1 * 03	HLA-DRB103 *	Lower frequency in mold-sensitive individuals, possibly protective.

* The asterisk separates the gene locus (e.g., A, B, DRB1) from the specific allele group and subsequent numbers, signaling high-resolution or ultra-high-resolution genetic identification (i.e., DNA sequencing) rather than older serological (e.g., antibody-based) techniques.

**Table 3:** Vasoactive and neurotoxic mast cell mediators.

Bradykinin; Carboxypeptidase A; Chymase; Corticotropin-releasing hormone (CRH); Hemokinin A; Histamine; IL-6; IL-17; IL-18; IL-33; Matrix metalloproteinase–(MMP-9); Neurotensin (NT); Nitric oxide; Osteopontin; Prostaglandin D_2_; Substance P (SP); TNF-α; Tryptase; Vascular endothelial growth factor (VEGF); Vasoactive intestinal peptide (VIP).

**Table 4:** Diagnostic markers for gut–brain inflammation.

**Blood**
Calprotectin; Gliadin; Histamine; Eosinophilic cationic protein; Eosinophilic-derived neurotoxin (EDN, RNase 2); Lysozyme; Matrix metalloproteinase-9 (MMP-9); Neurofilament light (NfL); Propionic acid; Secretory IgA; Tissue transglutaminase (tTG); Vascular endothelial growth factor (VEGF); Vasoactive intestinal peptide (VIP); Zonulin.
**Stool**
Calprotecin; Histamine; Eosinophilic cationic protein; Eosinophilic-derived neurotoxin (EDN, RNase 2).
**Urine (24 h collection cold)**
N-methylhistamine; Leukotriene E_4_; Prostaglandin F_2α_.

**Table 5:** Suggested dietary supplements for Autism Spectrum Disorders.

Main Target	Products	Actions
**Neuronal Health**	Folinic acid, calcium folinate = Leukovorin ^#^	Can bypass dysfunctional folate receptors. Does not require MTHFR-best in the presence of mutations (C677T)
	5-Methytetrahydrofolate = 5-MTHF ^#^	The active form of folate *
**Neuro-** **Inflammation**	Berberine	Antipathogenic, mast cell blocker
	*Bifidobacterium infantis*, *B. lactis*, *B. longum*	Reduce histamine, anti-inflammatory
	Butyrate	Strengthens gut–blood barrier, anti-inflammatory, mast cell blocker
	DAO (diamine oxidase) ^&^	Degrades histamine
	Luteolin + Quercetin (liposomal in olive pomace oil) ^@^	Anti-oxidant, anti-allergic, anti-inflammatory, mast cell and microglia blockers, neuroprotective
	Tetramethoxyluteolin ^+^	Anti-allergic, anti-inflammatory, mast cell, and microglia blocker
	Palmitoyl ethanolamide (PEA)	Anti-inflammatory, immune regulator
	Vitamin D3	Immune regulator
**Oxidative Stress**	Glutathione	Anti-oxidant
	N-Acetyl cysteine (NAC)	Increases glutathione, an antioxidant

^#^ VitalFolinic with 5-MTHF^®^; * still requires folate receptors to enter brain cells; ^&^ best acid-resistant or enteric-coated; ^@^ NeuroProtek^®^; ^+^ GentleDerm^®^.

## Data Availability

No new data were created or analyzed in this study. Data sharing is not applicable to this article.

## Abbreviations

The following abbreviations are used in this manuscript:

ASD	Autism Spectrum Disorder
BPA	Bisphenol A
BBB	Blood–brain barrier
Cpa3	Carboxypeptidase 3
CDC	Centers for Disease Control
CSF	Cerebrospinal fluid
HDAC3	Class I histone deacetylase 3
CTMC	Connective tissue MCs
CRH	Corticotropin-releasing hormone
DAO	Diamine oxidase
GI	Gastrointestinal
GBB	Gut–blood-barrier
HK-1	Hemokinin-1
HNMT	Histamine N-methyl transferase
hiPSCs	Human induced pluripotent stem cell
HLA	Human leukocyte antigen
ING-γ	Interferon γ
IL	Interleukin
LPS	Lipopolysaccharide
MRGPRX2	Mas-related G protein-coupled receptor X2
MCs	Mast cells
MCAS	Mast cell activation syndrome
MIGD	Metabolic index of gut dysfunction
MTHFR	Methylenetetrahydrofolate reductase
MMC	Mucosal MC
MMP-9	Matrix etalloproteinase-9
NGF	Nerve growth factor
NfL	Neurofilament light
NT	Neurotensin
HNMT	N-methyl transferase
NF-κΒ	Nuclear factor kappa-light-chain-enhancer of activated B cells
OCD	Obsessive–compulsive disorder
OTA	Ochratoxin A
PEA	Palmitoyl ethanolamide
PAMPs	Pathogen-associated molecular patterns
PST	Phenol sulfur transferase
PAF	Platelet activating factor
PCB	Polychlorinated biphenyl
PET	Positron emission tomography
PGD_2_	Prostaglandin D_2_
SARS	Severe acute respiratory syndrome
SCFAs	Short chain fatty acids
SP	Substance P
tTG	Tissue transglutaminase
TLR	Toll-like receptor
TNF	Tumor necrosis factor
VEGF	Vascular endothelial factor
VIP	Vasoactive intestinal peptide
